# Improvement of spinal cord injury symptoms by targeting the Bax/Bcl2 pathway and modulating TNF-α/IL-10 using Platelet-Rich Plasma exosomes loaded with dexamethasone

**DOI:** 10.3934/Neuroscience.2023026

**Published:** 2023-11-20

**Authors:** Naeimeh Akbari-Gharalari, Maryam Ghahremani-Nasab, Roya Naderi, Zeinab Aliyari-Serej, Mohammad Karimipour, Parviz Shahabi, Abbas Ebrahimi-Kalan

**Affiliations:** 1 Department of Neurosciences and Cognition, School of Advanced Medical Sciences, Tabriz University of Medical Sciences, Tabriz, Iran; 2 Department of Tissue Engineering, School of Advanced Medical Sciences, Tabriz University of Medical Sciences, Tabriz, Iran; 3 Neurophysiology Research Center, Cellular and Molecular Medicine Research Institute, Urmia University of Medical Sciences, Urmia, Iran; 4 Department of Physiology, School of Medicine, Urmia University of Medical Sciences, Urmia, Iran; 5 Department of Applied Cell Sciences, School of Advanced Medical Sciences, Tabriz University of Medical Sciences, Tabriz, Iran; 6 Department of Anatomical Sciences, Faculty of Medicine, Tabriz University of Medical Sciences, Tabriz, Iran; 7 Department of Physiology, Faculty of Medicine, Tabriz University of Medical Sciences, Tabriz, Iran; 8 Neurosciences Research Center, Tabriz University of Medical Sciences, Tabriz, Iran

**Keywords:** spinal cord injury, PRP-Derived exosome, dexamethasone, drug delivery, signaling pathways

## Abstract

Spinal cord injury (SCI) is a debilitating condition that results in impaired sensory and motor function due to the limited self-regenerative ability of the spinal cord. To address this issue, combination therapy has been proposed as an effective treatment strategy for SCI regeneration. In this study, Platelet-Rich Plasma (PRP)-derived exosomes loaded with dexamethasone were utilized in a mouse model of SCI compression. PRP-derived exosomes loaded with dexamethasone (Dex) were prepared using ultracentrifugation and sonication methods and were administered to the mice via intravenous injection. Following a four-week duration, behavioral assessments were administered to assess functional recuperation, and diverse metrics encompassing the expression of genes associated with apoptosis and antiapoptosis, serum cytokine concentrations and tissue sampling were subjected to thorough examination. The results of this study demonstrated that mice treated with PRP-derived exosomes loaded with Dex (ExoDex) exhibited altered levels of TNF-α and IL-10, along with decreased Bax and increased Bcl2 expression in comparison to the model group. Furthermore, intravenously injected ExoDex reduced the size of the lesion site, lymphocyte infiltration, vacuolation, cavity size and tissue disorganization while also improving locomotor recovery. We propose that the utilization of exosome-loaded Dex therapy holds potential as a promising and clinically relevant approach for injured spinal cord repair. However, further extensive research is warranted in this domain to validate and substantiate the outcomes presented in this study.

## 1. Introduction

Spinal cord injury (SCI) is a devastating event that ranks as a top cause of mortality worldwide [1]. SCI often leads to long-lasting impairments in sensory, motor and autonomic functions, profoundly affecting individuals' quality of life [2]. SCI involves both primary and secondary physical damage, with the initial injury causing acute bleeding and ischemia that serve as the starting point for subsequent damage. Therefore, limiting the lesion size in the early stages is critical for minimizing secondary damage [3],[4]. Currently, there are only a few effective treatments for neuroprotection and regeneration of the spinal cord after SCI [5],[6]. It is thus imperative to identify promising strategies to improve the outcomes of SCI.

Exosomes, the primary subclass of extracellular vesicles, are released by almost any type of cell into the extracellular environment [7]. These vesicles possess distinct biochemical and biophysical characteristics [8]. Due to their excellent delivery mechanism and ability to remain stable in the blood, exosomes can travel long distances within the body in both pathological and physiological situations [9]. Moreover, their lipid bilayer membrane and aqueous core enable them to transport both lipophilic and hydrophilic drugs [10],[11]. Exosomes can transmit signals between different species without displaying any species-specific characteristics, making them valuable in biomedicine, where they can be obtained from various species and modified [12],[13]. Exosomes play a crucial role in mitigating secondary damage by interacting with activated microglia and cytokines, thereby preventing the production of proinflammatory molecules [14],[15]. In the treatment of SCI, the primary strategy is to inhibit the proinflammatory environment, as supported by the correlation between the level of the cytokine IL-10 and the degree of motor functional recovery in SCI patients, as well as by the reduction in the inflammatory response of tumor necrosis factor (TNF-α) [15],[16]. Research has shown that exosomes can enhance neural function recovery by reducing the expression of the pro-apoptotic protein Bcl-2-associated X (Bax) and upregulating the anti-apoptotic protein B-cell lymphoma-2 (Bcl-2) [17]. Platelet-rich plasma-derived exosomes have demonstrated potential in the treatment of chronic wounds, primarily due to the growth factors they contain [18],[19]. However, the roles and existence of these exosomes require further investigation.

Glucocorticoid drugs are influenced by various mechanisms, including the inhibition of lipid peroxidation, which reduces cytokine expression and release, thus impeding inflammation [20]. Dexamethasone (Dex), a glucocorticoid analog, exerts multifaceted anti-inflammatory effects. Specifically, Dex suppresses phagocytosis and reduces lysosomal secretion by decreasing lysosomal elastase, prostaglandins, arachidonic acid, leukotriene B4 and thromboxane B2 [21],[22]. Dex also inhibits macrophage activation induced by oxidized lipoprotein, which coincides with a reduction in the rates of granulocyte/macrophage colony-stimulating factor [23],[24]. Moreover, Dex mitigates macrophage toxicity by inhibiting nitric oxide production and inducible nitric oxide synthase [23]. However, the administration of Dex in excessive doses may lead to unwanted side effects such as hyperglycemia, adrenal insufficiency, osteoporosis, hypertension and diabetes mellitus [25]. Given the properties of exosomes and dexamethasone, PRP-derived exosomes loaded with Dex (Exo-Dex) have the potential to enhance treatment efficacy and reduce systemic adverse effects [26].

In this study, we investigated the effects of PRP-derived exosomes, both with and without dexamethasone, on histological, molecular and motor function alterations in a mouse model of SCI. The study focused on exosome-derived content, including lipids, cytokines, enzymes, immunoregulatory and trophic proteins and growth factors.

## 2. Materials and methods

### 2.1. Isolation of human PRP-derived exosomes by ultracentrifugation

In this study, 100 mL of venous blood was collected from twelve healthy male donors aged between 30 and 40 years to prepare PRP. The samples were incubated at room temperature for 30 minutes before being centrifuged at 300×g for 10 minutes. The supernatant was then centrifuged at 1800×g for 10 minutes to obtain the plasma fraction according to the methods of Torreggiani et al. [27]. The PRP was mixed with calcium gluconate solution and incubated at 37 °C for 30 minutes, and the resulting clot was centrifuged at 2000×g for 10 minutes. Next, the supernatant was mixed with calcium gluconate solution and incubated at 37 °C for 2 hours before being centrifuged at 2000×g for 10 minutes. The supernatant was then subjected to ultracentrifugation (OPTIMA TLX 361544) at 100000×g for 2 hours at 4 °C, resulting in the sedimentation of the intended exosomes. The exosomes were subsequently eluted with PBS and stored frozen at −80 °C.

### 2.2. Characterization of exosomes isolated from PRP

The study employed dynamic light scattering (DLS) using the Malvern Zetasizer Nano ZS in the UK to determine the size distribution and zeta potential of the exosomes at a temperature of 25 °C. The concentration of total protein in the sample was measured using the Bradford protein assay, which involves reading the absorbance at 595 nm with a spectrophotometer (CE7250). Furthermore, the size and morphology of the exosomes were analyzed through field emission scanning electron microscopy (MIRA3 FEG-SEM Tescan).

### 2.3. Encapsulation and release kinetics of Dex

In the present investigation, the sonication method previously described by Wan et al. [28] was utilized to encapsulate dexamethasone into exosomes. Specifically, 12 µg of Dex was mixed with 20 µg of exosomes in a total volume of 100 µl at 37 °C for 20 minutes. The mixture was then subjected to sonication using a water bath sonicator (WUC-D10H) with the following parameters: 20% amplitude, 6 cycles of 30 seconds on/off for 3 minutes, with a 2-minute cooling period between each cycle. After sonication, the compound was incubated at 37 °C for 60 minutes and then ultracentrifuged at 100000×g for 2 hours to allow the exosomes to settle. The amount of Dex loaded into exosomes was quantified using UV spectrophotometry by analyzing the absorbance wavelength of the supernatant at 242.5 nm.

To determine the quantity of drug released from exosomes, spectrophotometry was employed over a period of 72 hours. The exosomes were dissolved in PBS and placed in a 10 kDa dialysis bag containing PBS medium, after which the absorption wavelength was measured.

### 2.4. Animals and surgical procedure

We utilized sixty healthy adult female BALB/c mice with an average weight of 24 ± 2 g and an age range of 8–10 weeks. These mice were provided by Tabriz University of Medical Science and were kept under standard conditions. The protocols for animal experimentation were approved by the Experimental Animal Care and Ethics Committee of the university (registration no. IR.TBZMED.AEC.1400.009). The study consisted of six groups of mice, each with ten individuals: Control, laminectomy, SCI model, Exo, Dex and ExoDex.

We induced compression SCI in mice, which were anesthetized with ketamine (80 mg/kg) and xylazine (20 mg/kg) via intraperitoneal injection. Prior to the surgery, vitamin A ointment was applied to the eyes to prevent corneal dryness. The target region was shaved and sterilized with 70% alcohol and betadine, after which a longitudinal incision was made on the lower half of the thoracic segments using a surgical blade. The fascia and paravertebral muscles were removed from the T9-T11 vertebrae, and the T10 vertebral lamina bone was carefully exposed without damaging the spinal cord. This procedure, known as a laminectomy, exposed the dorsal part of the T10 spinal cord. The sides of the spinal cord were then compressed using specialized forceps with a tooth spacing of 0.3 mm for 15 seconds. After the surgery, the skin was sutured, and the mice were kept warm until they regained consciousness. The mice were kept in separate cages containing thick blankets during the experimental period to minimize infections. The cages were washed daily to maintain a clean environment. Following the induction of SCI, the mice lost their voiding reflex, and their bladders were emptied twice a day until the reflex returned.

The different groups of mice were treated with various substances via tail vein injection. The first group received exosomes (0.4 mg/kg), which consisted of 10 µg of exosomes in 100 µl of PBS and were administered every three days, starting one hour after modeling, for a total of 28 days. The second group received Dex (0.5 mg/kg), which was dissolved by mixing 12 µg of Dex powder in 100 µl of PBS and was administered once daily for five consecutive days. Furthermore, the third group was administered a combination of Exo-Dex at three-day intervals. In addition, the mice were given daily intraperitoneal injections of normal saline (100 ml/kg/day) to prevent dehydration and ceftriaxone (100 mg/kg/day) to prevent postoperative infections for seven days. Furthermore, the mice were given subcutaneous ketoprofen (0.5 mg/kg/day) for three days to provide analgesia.

### 2.5. BBB locomotor test

The recovery of hind limb motor skills in BALB/c mice was assessed using the Basso, Beattie and Bresnahan (BBB) open-field locomotor scale, which was developed by Basso et al. [29]. This scale evaluates the locomotor functions of the lower limb on a 21-point rating system, with a score of 0 indicating complete paralysis and 21 indicating normal status. Three hours after the surgery, when the animals had regained consciousness, they were placed individually in an open field and allowed to move freely for five minutes. Two independent observers who were blinded to the treatment groups assessed the mice's performance for up to four weeks to evaluate the recovery of hind limb motor skills using the BBB scale.

### 2.6. In vivo fluorescence tracing of exosomes

To visualize exosomes, a red DiI (1,1′-dioctadecyl-3,3,3′,3′-tetramethylindocarbocyanine perchlorate) lipid stain was used to fluorescently label them. Exosomes were incubated with the dye at a ratio of 400:1 in volume for 30 minutes at 37 °C [30]. The concentration of exosomes used for staining was 1 µg/µL. After staining, the exosomes were purified by ultracentrifugation to remove the unbound dye. To investigate exosome homing, DiI-labeled PRP exosomes were injected into the tail vein of three BALB/c mice and diluted in 100 µL of sterile PBS. After 24 hours, the mice were sacrificed, and their spinal cords were extracted. Cryosections of the damaged regions of the longitudinally and transversely injured spinal cord tissues were prepared and analyzed using fluorescence microscopy to quantify the fluorescence intensity of the exosomes.

### 2.7. Tissue processing and histopathological studies

To assess the extent of lymphocyte infiltration, hematoma formation, vacuolation, cavity/lesion size and tissue disorganization in different experimental groups, spinal cord tissue samples were collected from the T8-T12 region 28 days postinjury and subjected to hematoxylin-eosin (H&E) staining. The mice were anesthetized deeply and subsequently sacrificed by intracardiac perfusion with freshly prepared 10% formaldehyde. The fixed tissue samples were processed through a series of steps, including washing, dehydration, clearing, paraffinization and embedding in paraffin. Longitudinal sections of 5 µm thickness with 10 slice intervals were prepared using a microtome and mounted onto glass slides. These sections were subjected to H&E staining, and the resulting images were analyzed using Kview software with two blinded observers scoring each criterion on a scale of 0 to 4 (0 indicating “no” and 4 indicating “high”). The images were then subjected to morphometric analysis using ImageJ software.

### 2.8. Measurements of serum cytokine levels

Following collection, a cardiac blood sample was immediately subjected to centrifugation at 10000×g for 5 minutes. The resulting serum samples were stored at −70 °C until cytokine measurement. The concentrations of two cytokines, TNF-α and IL-10, were quantified in the serum samples using commercially available enzyme-linked immunosorbent assay (ELISA) kits from R&D Systems, Minneapolis, USA.

### 2.9. RNA extraction and cDNA synthesis

Total RNA was extracted from the spinal cord using TRIzol reagent (Gibco, USA), and complementary DNA (cDNA) was synthesized from the total RNA using a cDNA synthesis kit (Pars Tous, Iran) according to the manufacturer's instructions.

### 2.10. Quantitative real-time PCR

Complementary DNA (cDNA) synthesized from the extracted total RNA was used to detect the expression of the Bax and Bcl-2 genes using specific oligonucleotide primers (Table 1).

**Table 1.** Primer set list.

Gene	Primer Sequence^1^
Bax	F: AGGGTTTCATCCAGGATCGAG/R: TCCACGTCAGCAATCATCCTC
Bcl-2	F: GCCTCTTCACCTTTCAGCATTG/R: TTCCCCGTTGGCATGAGATG
β-actin	F: TACAGCTTCACCACCACAGC/R: ATGCCACAGGATTCCATACC

^1^ Sequences were derived from NCBI (www.ncbi.nlm.nih.gov).

The reaction mixture comprised 10 µl, containing 5 µl of prime Q-Master Mix with SYBR Green I (Pars Tous, Iran), 1.1 µl of cDNA, 1 µl of forward and reverse primers and 2.9 µl of sterile deionized water, according to the manufacturer's instructions.

Real-time-PCR was performed using the LightCycler-FastStart DNA Master SYBR Green system (Roche Molecular Biochemicals, Mannheim, Germany) with a 15-minute preincubation at 95 °C, followed by 55 cycles of 60 seconds at 58 °C (denaturation) and 10 seconds at 95 °C (annealing). The products were analyzed for a melting curve using the light cycler system to avoid nonspecific product amplification. The β-actin gene with the same annealing temperature was used as an internal control for housekeeping gene expression. All reactions were performed in duplicate.

### 2.11. Statistical analysis

The statistical analysis employed for this study involved the utilization of GraphPad Prism software (version 9.3.1, USA) for data analysis and graphical representation. The serum parameters, modifications in spinal cord tissue and gene expression are depicted as the mean ± standard error of the mean (SEM). To ascertain significant distinctions among the three distinct groups, a two-way analysis of variance (ANOVA) was conducted, followed by Tukey's post hoc test. Semiquantitative data derived from histological evaluations were characterized as the median (ranging from the minimum to the maximum) and subjected to analysis using the Kruskal-Wallis test. In instances where notable effects were observed, the Mann-Whitney test was applied for post hoc analysis. Statistical significance was established for p-values less than 0.05.

**Figure 1.**
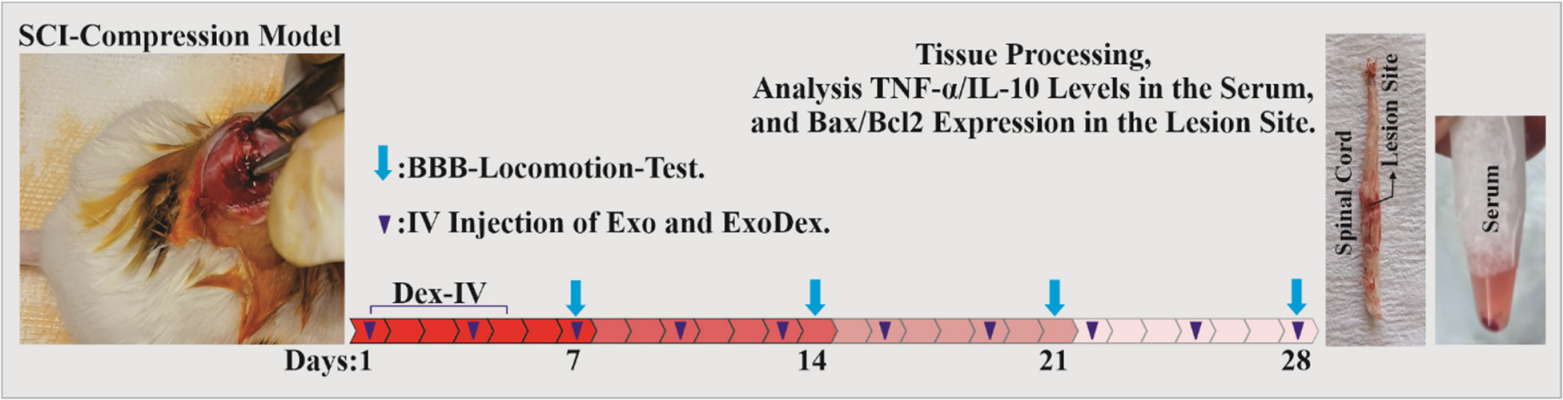
This visual representation delineates the chronological sequence of events observed throughout the course of the investigation.

## 3. Results

### 3.1. Characterization of exosomes

**Figure 2.**
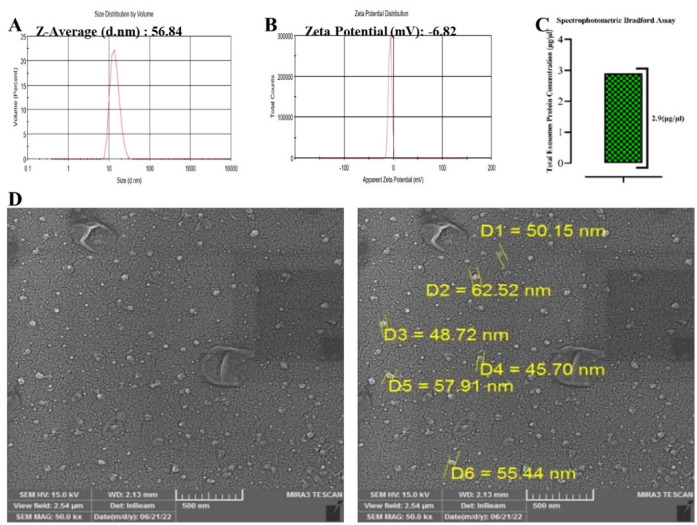
Characterization of exosomes derived from PRP. A. The size distribution of the exosomes. B. Zeta potential result. C. Total protein levels of the exosomes. D. SEM view of the morphology and size of the exosomes.

Exosomes were isolated from PRP obtained from healthy human donors. The size distribution and zeta potential of the exosomes were determined using dynamic light scattering (DLS). The pure exosomes had a uniform size distribution, with a mean diameter of 56.84 nm (Figure 2-A) and a zeta potential of approximately −6.82 mV (Figure 2-B), which was consistent with the DLS measurements. The amount of exosomal protein was quantified using a spectrophotometric Bradford assay, and 2.9 µg of protein was detected in 1 µl of exosome solution (Figure 2-C). The morphology of the exosomes was examined using scanning electron microscopy (SEM), which confirmed the results obtained from DLS (Figure 2-D). The exosomes were then stored at −80 °C for further use.

### 3.2. Dex loading and release rates

**Figure 3.**
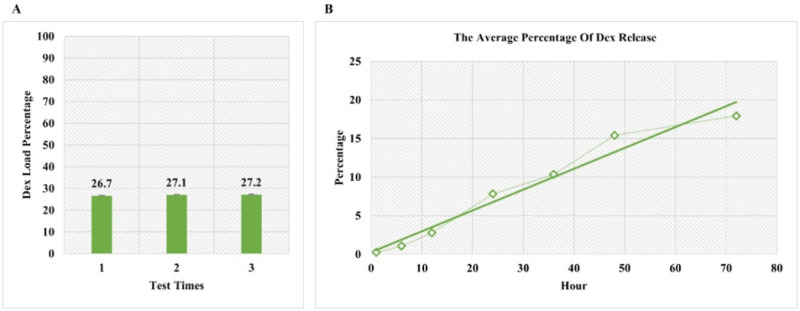
A. The amount of Dex encapsulated in exosomes. B. The percentage of Dex released from exosomes.

Following the sonication method for loading Dex into exosomes, the amount of Dex encapsulated was measured using spectrophotometry at the intended λ max, and the Dex incorporation rate was found to be approximately 27% (Figure 3-A). Previous studies have shown that the surface proteins of exosomes remain unchanged after sonication-mediated Dex loading [26]. The amount of Dex released from the exosomes was evaluated using spectrophotometry over a 72-hour period, and it was observed that approximately 17.92% of the incorporated drug was released during this time (Figure 3-B).

### 3.3. BBB locomotion scores

**Figure 4.**
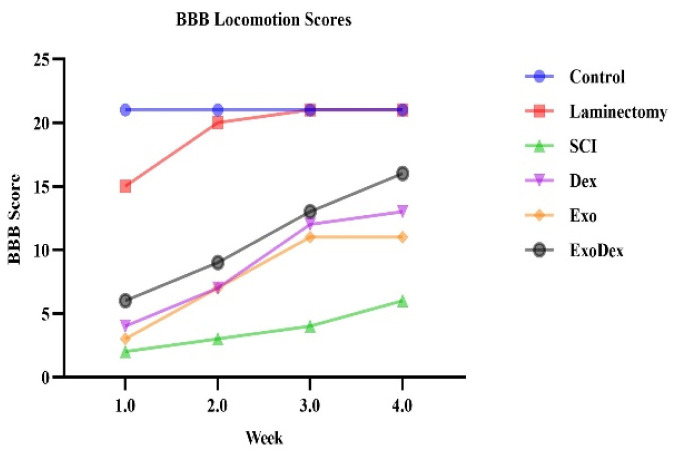
Illustrates the progression of motor function improvement over a four-week period.

**Figure 5.**
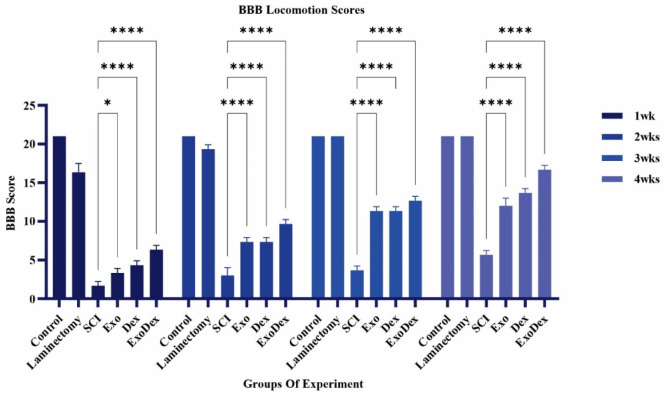
The scores were compared between the various experimental groups (*P = 0.0136, ****P < 0.0001).

The BBB test was administered three hours after surgery and then once a week, and the average scores are presented in Table 2. The trend of improvement in motor functions over four weeks is depicted (Figure 4), while a comparison is made between the scores of different experimental groups (Figure 5). The ExoDex group showed the most recovery compared with the other spinal cord injury groups. Then, the Dex and Exo groups improved the most (P < 0.0001). The SCI group received the lowest score. The Laminectomy group reached normal motor function in the second week.

**Table 2.** BBB locomotion scores for all groups.

Week					
		**1**	**2**	**3**	**4**
Groups	**Control**	21	21	21	21
	**Laminectomy**	16.3	19.3	21	21
	**SCI**	1.6	3	3.6	5.6
	**Exo**	3.3	7.3	11.3	12
	**Dex**	4.3	7.3	11.3	13.6
	**ExoDex**	6.3	9.6	12.6	16.6

### 3.4. Extensive diffusion of exosomes over long distances

**Figure 6.**
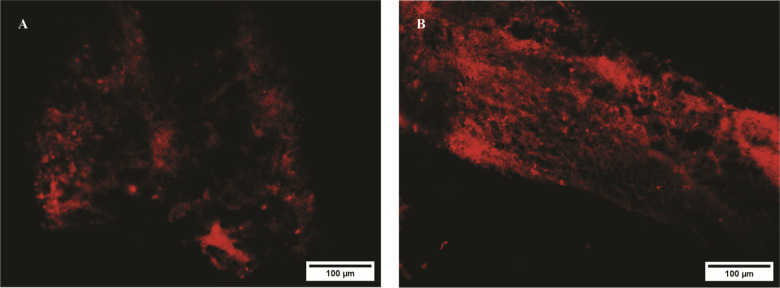
Localization of DiI-labeled exosomes. A. Cross-sections of the spinal cord tissue. B. Longitudinal section of the spinal cord tissue.

To investigate whether exosomes derived from PRP can be internalized into the spinal cord following injury, the exosomes were labeled with the red fluorescent dye DiI. Twenty-four hours after intravenous injection of DiI-labeled exosomes, their localization was examined under a fluorescence microscope. The results demonstrate that the exosomes were able to gradually spread from the injection site to other regions of the spinal cord (Figure 6).

### 3.5. Histological assessments

**Figure 7.**
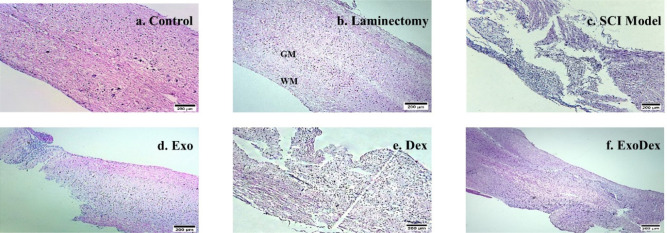
These images (a, b, c, d, e and f) illustrate examples of each group stained with H&E. b. Gray matter (GM) and white matter (WM) are two distinct areas of neural tissue.

**Figure 8.**
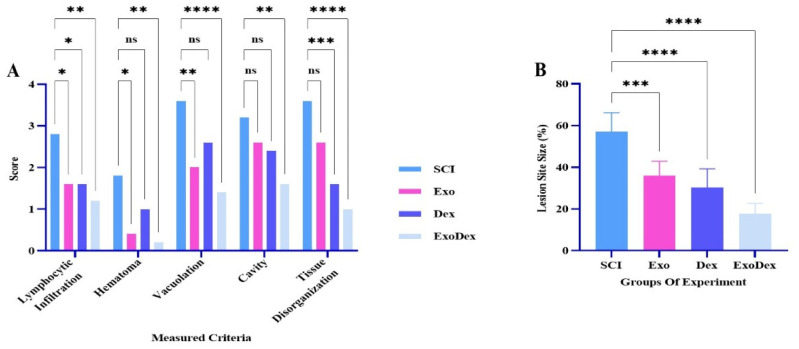
A. Analysis of the scores for the evaluated criteria. In the Exo group, there was a decrease in cavity size (P = 0.5222) and tissue disorganization (P = 0.1108), but the change was not significant. However, there was a substantial reduction in lymphocyte infiltration (*P = 0.0374), hematoma (*P = 0.0106) and vacuolation (**P = 0.0026). In the Dex group, there was a considerable reduction in tissue disorganization (***P = 0.0001) and lymphocyte infiltration (*P = 0.0374), and although there was a decrease in hematoma (P = 0.2690), vacuolation (P = 0.1108) and cavity (P = 0.2690), it was not significant. In the ExoDex group, all criteria showed a significant decrease (**P = 0.0026, ****P < 0.0001). B. Comparison of the average percentage of the lesion area among different groups. In all three treatment groups, the damaged area was significantly reduced compared to that in the model group. The ExoDex group (****P < 0.0001) exhibited a greater reduction compared to the Exo (***P < 0.0002) and Dex (****P < 0.0001) groups.

In this study, H&E staining was used to analyze histological sections of tissue samples taken 28 days postinjury, with the aim of evaluating the morphology of the lesion. Examples of images from each group are provided (Figure 7). The scores of the measured criteria for histopathological analysis in the different experimental groups are shown (Figure 8-A). The results showed a substantial decrease in lymphocyte infiltration in all groups. Hematoma formation was significantly reduced in the Exo and ExoDex groups but not in the Dex group. Similarly, vacuolation was markedly reduced in the Exo and ExoDex groups but not in the Dex group. Cavity size was reduced in all groups, but the reduction was only significant in the ExoDex group. Tissue disorganization was significantly reduced in the Dex and ExoDex groups but not in the Exo group.

Furthermore, the lesion site size (%) demonstrated that the size of the lesion site decreased significantly in all treated groups compared to the model group (Figure 8-B). These findings suggest that the treatment options under investigation in this study had a positive impact on lesion morphology and size, with the ExoDex group exhibiting the most significant improvements across all criteria.

### 3.6. Levels of serum cytokines

**Figure 9.**
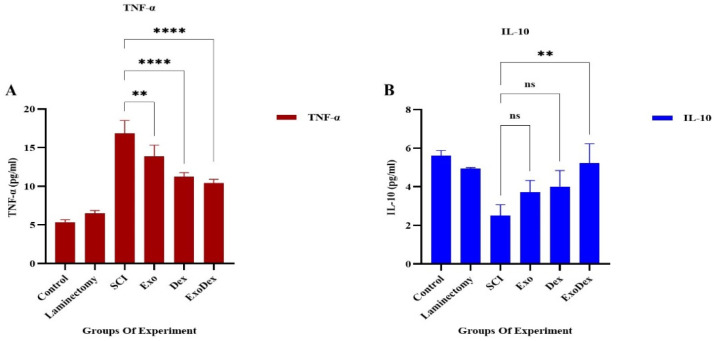
A. The levels of TNF-α in the Exo (**P = 0.0043), Dex (****P < 0.0001) and ExoDex (****P < 0.0001) groups decreased significantly compared to those in the SCI group. B. The levels of IL-10 increased in all three treatment groups, and the increase was not substantial in the Exo (P = 0.5262) and Dex (P = 0.2968) groups. However, in the ExoDex (**P = 0.0084) group, there was a significant increase in the level of IL-10.

The levels of TNF-α and IL-10 in the serum were measured using ELISA. The results showed that in the Exo, Dex and ExoDex groups, the levels of TNF-α were significantly lower than those in the SCI group (Figure 9-A). The level of IL-10 was increased after administration of Exo and Dex, but the difference was not significant when compared to the SCI group. However, in the ExoDex group, there was a substantial increase in the level of IL-10 (Figure 9-B).

### 3.7. Analysis of Bax/Bcl2 expression at the site of spinal cord lesions

**Figure 10.**
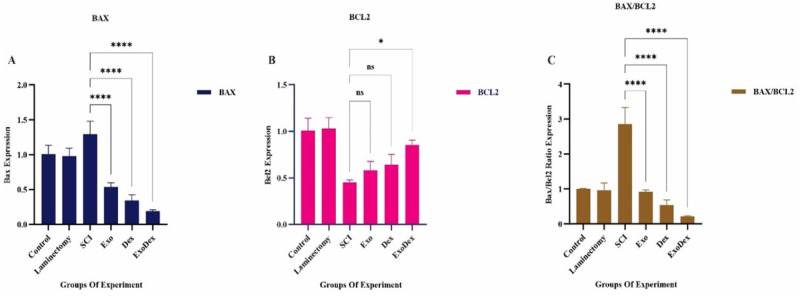
A. The treatment groups, Exo, Dex and ExoDex, significantly decreased the expression of the apoptotic Bax gene compared to the SCI group (****P < 0.0001). B. The Exo (P = 0.8983) and Dex (P = 0.6636) treatments increased the expression of the antiapoptotic Bcl2 gene, but the difference was not significant. However, the ExoDex (*P = 0.0297) group substantially increased the expression of this gene. C. When comparing the expression ratio of Bax to Bcl2 in all groups, there was a significant decrease compared to the SCI group (****P < 0.0001).

SCI induces an increase in apoptotic Bax gene expression compared to the control and laminectomy groups. However, in the Exo, Dex and ExoDex treatment groups, the expression of this gene was significantly decreased compared to that in the SCI group (Figure 10-A). The results indicate that these treatments also reduce apoptosis in healthy mice. After injury, the expression of the antiapoptotic Bcl2 gene decreased, and while the Exo and Dex treatment groups increased the expression of this gene, the difference was not significant. However, the ExoDex group substantially increased the expression of the Bcl2 gene (Figure 10-B). When comparing the ratio of apoptotic to antiapoptotic gene expression, it was observed that all three treatment groups had a significant decrease in this ratio compared to the model group (Figure 10-C).

## 4. Discussion

SCI is a complex pathological process that leads to tissue damage. Proinflammatory cytokines, such as TNF-α, play a crucial role in this destruction by inducing apoptosis, oxidative stress and inflammation.

TNF-α activates nuclear factor-kappa B, a transcription factor that regulates genes involved in inflammation and the immune response. This activation results in an increase in pro-inflammatory cytokines and chemokines, which exacerbates the inflammatory response. TNF-α also modulates oxidative stress pathways, leading to the production of reactive oxygen species and cell death while inhibiting antioxidant enzyme activity. Furthermore, TNF-α induces proapoptotic genes and caspase-mediated cell death, affecting apoptosis signaling pathways. In contrast, anti-inflammatory cytokines such as IL-10 play a pivotal role in SCI by controlling the immune response and promoting tissue healing. Activation of the Janus kinase/signal transducer and activator of transcription (JAK/STAT) pathway by IL-10 leads to the production of anti-inflammatory genes. Additionally, IL-10 suppresses proinflammatory cytokines such as TNF-α and IL-1β and promotes anti-inflammatory cytokines such as IL-4 and IL-13, thereby regulating the function of immune cells. Furthermore, IL-10 modifies apoptosis signaling pathways in SCI by suppressing the expression of proapoptotic genes such as Bax and increasing antiapoptotic genes such as Bcl-2 [31].

As previously mentioned, the balance between Bax and Bcl2 expression and the activity of cytokines, such as TNF-α and IL-10, is critical in determining apoptotic cell death and inflammation in SCI. Bax is a proapoptotic protein that induces apoptotic cell death, while Bcl2 is an antiapoptotic protein that promotes cell survival. Bax expression is regulated by various signaling pathways, including the p53 pathway, oxidative stress pathways and cytokine signaling. Conversely, Bcl2 expression is regulated by different pathways, including the PI3K/Akt pathway, oxidative stress pathways and growth factor/cytokine signaling [32]. Our research has shown that the expression of Bax and Bcl2 and the activity of cytokines, such as TNF-α and IL-10, play pivotal roles in determining apoptotic cell death and inflammation in SCI (Figures 9, 10). This study aimed to investigate the impact of pure exosomes, Dex and exosomes containing Dex on SCI, and therefore, the characteristics of exosomes are significant to our findings.

Exosomes have been shown to play crucial roles in regulating the expression of inflammatory cytokines, such as TNF-α and IL-10, in the injured spinal cord. Exosomes derived from bone marrow mesenchymal stem cells (BMSCs) or human umbilical cord mesenchymal stem cells (hUC-MSCs) were found to reduce TNF-α expression and increase IL-10 expression in rat models of SCI. Moreover, exosomes can modulate the expression of pro-apoptotic Bax and anti-apoptotic Bcl2, suggesting their potential role in regulating apoptosis [33]. Exosomes are known to contain various bioactive molecules, including miRNAs, that may mediate their effects. For instance, miR-146a targets genes involved in the regulation of immune responses, modulating the production of proinflammatory cytokines and inhibiting the activation of the NF-kB pathway, leading to an anti-inflammatory phenotype. Additionally, miR-146a regulates the expression of genes involved in apoptosis. MiR-124a, which is highly expressed in the central nervous system, regulates neurogenesis, neural differentiation and synaptic plasticity. Exosomes containing miR-124a have the ability to regulate gene expression in other cells, such as astrocytes and microglia [34],[35]. This property of exosomes, combined with the anti-inflammatory and immunosuppressive properties of Dex, makes it a promising therapeutic approach for SCI. Dex has been shown to improve secondary injury and promote tissue repair in SCI patients by reducing proinflammatory cytokine expression and increasing anti-inflammatory cytokine expression.

Dex has also been found to decrease Bax expression and increase Bcl2 expression in a model of SCI [36]. However, Dex and other glucocorticoids can cause side effects such as immunosuppression, osteoporosis and glucose intolerance, so their use in SCI treatment must be carefully considered and monitored to minimize adverse effects. The combination of exosomes and Dex may enhance therapeutic efficacy by protecting Dex from degradation, delivering it specifically to target cells, and reducing the occurrence of side effects associated with Dex administration. This approach could also lower the needed drug dosage, making it a promising treatment option for SCI. We developed the Exo-Dex delivery system, which substantially improved motor function recovery in SCI by suppressing the expression of the apoptotic Bax gene and increasing the expression of the antiapoptotic Bcl2 gene (Figure 10). This effect was also found to reduce TNF-α levels and upregulate IL-10 levels in serum, providing further evidence of its effectiveness (Figure 9). Tissue analysis revealed improvements in the treated groups compared to the model group, as evidenced by a reduction in vacuolation, cavity size, lymphocyte infiltration, hematoma and tissue disorganization (Figure 8). This drug delivery system has several notable advantages for treating SCI. First, it can cross the blood–spinal cord barrier (BSCB), allowing for targeted delivery to the lesion site. Second, it does not cause secondary injury to the spinal cord, which is a common concern with other treatment methods. Third, the system is nonimmunogenic, meaning it does not elicit an immune response from the body. Last, the system reduces the needed dosage of Dex and its associated side effects. One of the primary challenges facing the clinical translation of exosome-based therapies is the sourcing of exosomes.

However, previous studies have shown that exosomes from various sources can be loaded with functional cargos using CP05, allowing them to serve as effective delivery vehicles. Exosomes derived from PRP are a promising source for this purpose, as they contain various membrane proteins, including CD63, and lack cancer-stimulating activities [37]. Moreover, PRP-derived exosomes are readily available and avoid immunological reactions associated with foreign exosomes. Additionally, exosomes derived from PRP are generated at higher yields and are of a preferable size compared to those from cell lines, facilitating their intracellular delivery. In this study, we utilized exosomes from PRP as delivery vehicles for Dex, demonstrating their potential as an effective therapeutic intervention for SCI. Therefore, PRP is a readily available source of exosomes that can be effectively used as drug delivery vehicles for SCI therapy. Exosomes derived from PRP are abundant and can be easily obtained through a simple and minimally invasive procedure. The unique properties of exosomes, including their ability to cross biological barriers, target damaged tissues and modulate immune responses, make them an attractive option for delivering therapeutic agents, such as Dex, to the site of SCI. The incorporation of Dex into exosomes can enhance their therapeutic efficacy. Dex is a clinically approved anti-inflammatory drug that is commonly used for SCI treatment. However, it can cause adverse effects. By incorporating Dex into exosomes, the drug can be targeted specifically to the site of injury and reduce the needed dosage, thereby minimizing potential side effects. Moreover, the use of exosomes as delivery vehicles for Dex can synergize with the inherent therapeutic potential of exosomes themselves. Exosomes possess inherent therapeutic properties, including their anti-inflammatory and immunomodulatory effects and their ability to promote tissue repair and regeneration. These properties can further enhance the therapeutic potential of exosome-based delivery systems for SCI. The fact that exosomes have demonstrated no adverse effects makes them attractive candidates for preclinical trials. In addition, exosomes are highly customizable and their cargo can be easily modified with functional molecules, such as miRNAs, to achieve targeted therapy.

**Figure 11.**
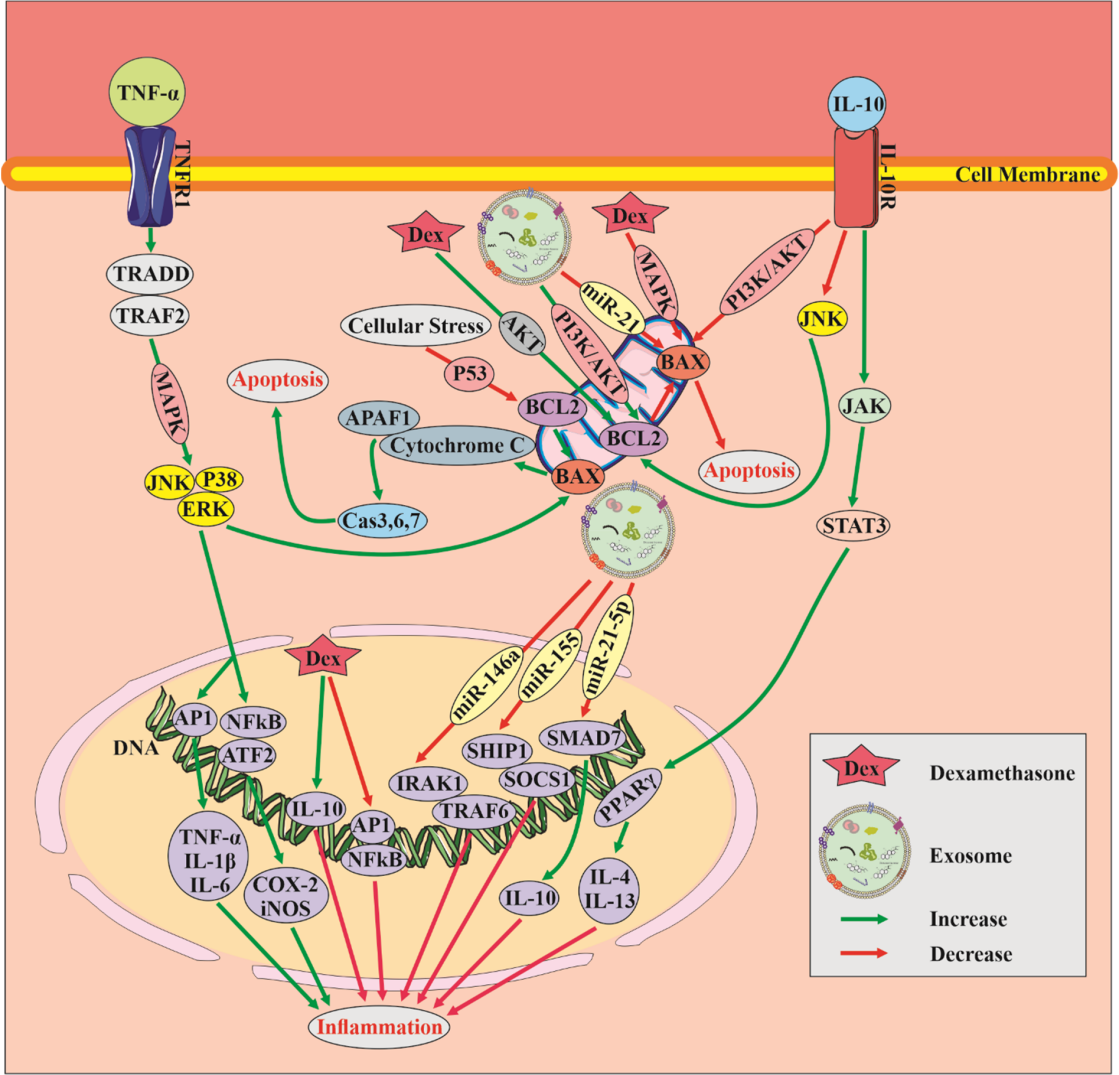
Investigation of The Signaling Pathways Regulating Apoptosis and Inflammation in Spinal Cord Injury. This illustration demonstrates the crucial role of Bax and Bcl2, as well as the inflammatory cytokines TNF-α and IL-10, in apoptotic and inflammatory processes. Additionally, the pathways influenced by exosome and dexamethasone treatments to reduce inflammation and apoptosis are clearly emphasized, implying their use in this research study.

In the context of our research study, Figure 11 serves as a pivotal visual representation that elucidates the intricate interplay of signaling pathways and the molecular mechanisms underlying the actions of key regulatory molecules in the regulation of apoptosis and inflammation. It provides a comprehensive view of the processes that occur when cytokines IL-10 and TNF-α bind to their respective receptors, initiating cascades of intracellular events that intersect with the pro-apoptotic protein Bax and the anti-apoptotic protein Bcl2. These interactions orchestrate significant alterations in cellular function, impacting both apoptosis and inflammation. The various signaling pathways through which both exosomes and dexamethasone demonstrate efficacy in mitigating inflammatory processes and apoptosis highlight the promising synergistic potential of these therapeutic agents. Dexamethasone, for instance, effectively reduces apoptosis by inhibiting the MAPK pathway and activating the Akt pathway, thus providing a dual antiapoptotic mechanism. On the other hand, exosomes exert their influence by suppressing Bax expression through miR-21 and enhancing Bcl2 levels via the PI3K/Akt pathway, leading to a suppression of apoptosis through the convergence of these two distinct pathways. Moreover, both dexamethasone and exosomes collaboratively modulate inflammation. Dexamethasone's anti-inflammatory effect is achieved by elevating the levels of IL-10 while concurrently inhibiting the NFĸB and AP1 signaling pathways. In contrast, exosomes harness the regulatory power of various miRNAs to temper the inflammatory response.

Overall, the use of exosomes as delivery vehicles for dexamethasone represents a highly promising therapeutic strategy for SCI. The integration of these two components, as demonstrated in Figure 11, showcases their complementary actions, offering immense potential for preclinical and clinical trials. This combined approach holds significant promise for advancing the treatment and management of SCI, warranting further investigation and development in the field of SCI research.

## 5. Conclusion

In conclusion, exosomes possess unique features that make them an attractive method for delivering therapeutic drugs, such as Dex, directly to the site of injury. The incorporation of Dex into exosomes has the potential to enhance the treatment of spinal cord injury symptoms by targeting the Bax/Bcl2 pathway and modulating TNF-α/IL-10. The intrinsic therapeutic properties of exosomes make them an appropriate choice for preclinical and clinical studies, which could further improve the efficacy of exosome-based delivery systems for SCI treatment.
